# Cochlear implantation outcomes in children with multiple disabilities: a topic that’s worth revisiting

**DOI:** 10.1016/j.bjorl.2024.101423

**Published:** 2024-03-21

**Authors:** Goh Bee-See, Nur Af’Idah Mohd Zulkefli, Asma Abdullah, Cila Umat, Norazlin Kamal Nor, Juriza Ismail, Stephen J. O’Leary

**Affiliations:** a Department of Otorhinolaryngology-Head and Neck Surgery, Faculty of Medicine, Universiti Kebangsaan Malaysia (UKM), Kuala Lumpur, Malaysia; b Hospital Canselor Tuanku Muhriz, Jalan Yaacob Latif, Bandar Tun Razak, Kuala Lumpur, Malaysia; c Center of Ear, Hearing and Speech (HEARS), Faculty of Health Sciences, Universiti Kebangsaan Malaysia (UKM), Kuala Lumpur, Malaysia; d Center for Rehabilitation & Special Needs Studies, Faculty of Health Sciences, Universiti Kebangsaan Malaysia (UKM), Kuala Lumpur, Malaysia; e Department of Paediatric & Child Development Centre (CDC), Faculty of Medicine,Universiti Kebangsaan Malaysia, (UKM), Kuala Lumpur, Malaysia; f Department of Surgery (Otolaryngology), University of Melbourne, and the Cochlear Implant Clinic, Royal Victorian Eye and Ear Hospital, Melbourne, Australia; g University of Melbourne, and the Cochlear Implant Clinic, Royal Victorian Eye and Ear Hospital, Department Surgery (Otolaryngology), Melbourne, Australia

**Keywords:** Cochlear implant, Children, Global developmental delay, Audiological outcome, Quality of life

## Abstract

**Objectives:**

To determine the benefits of cochlear implantation in hearing loss children with multiple disabilities (MD) in terms of auditory outcomes, speech performance, and their quality of life.

**Methods:**

This was a cross sectional study from January 2019 to December 2020 in which thirty-one children with hearing loss and multiple disabilities were evaluated. Their improvement in auditory and speech performances were assessed using Categories of Auditory Performance version II (CAP-II) and the Speech Intelligibility Rating (SIR) scales. The assessment was done at 6-month intervals, with the baseline evaluation done at least six months after activation of the implant. Parents were asked to fill the Parents Evaluation of Aural/Oral Performance of Children (PEACH) diary and Perceived Benefit Questionnaire (PBQ) to evaluate the child’s quality of life.

**Results:**

All 31 children have Global Developmental Delay (GDD), with 11 having an additional disability. Both mean CAP-II and SIR scores showed significant improvement with increased hearing age (*p* < 0.05) after 6-month intervals. In addition, 20 out of 31 children (64.5%) have achieved verbal communication after implantation. The mean PEACH score in quiet was significantly better than in noise (*p* = 0.007) and improved with the increased of hearing age. The majority of parents (96%‒100%) perceived a cochlear implant as beneficial to their child in terms of auditory response, awareness, interaction, communication, and speech development.

**Conclusions:**

Cochlear implantation had shown benefits in children with multiple disabilities. Outcome measures should not only focus on auditory and speech performances but the improvement in quality of life. Hence, individualized each case with realistic expectation from families must be emphasized in this group of children.

**Level of evidence:**

Level 3.

## Highlights

• Hearing loss children with multiple disabilities benefits from cochlear implantation.
• Communications improved with increasing duration of device usage.
• Cochlear implant is beneficial in reducing family’s stress level.

## Introduction

According to World Report on Hearing 2021, 1.5 billion people worldwide suffered from Hearing Loss (HL) globally, with 430 million having moderate to severe HL, and this number is expected to almost double by 2050.^1^ From this, 34 million are children, and it is well known that HL in children will implicate their language and speech development. Congenital HL can be due to genetic factors responsible for 50% of the neonatal HL (syndromic 15% and non-syndromic 35%), and intrauterine infections, which can be bacterial viral, or parasitic origins.^1^ Viral infections are responsible for 40% of non-genetic congenital HL with Cytomegalovirus (CMV) as the most common causes of HL, affecting 14% of infants. From these, 3%‒5% suffer from bilateral moderate to profound HL.^1^

Cochlear Implant (CI) has long been accepted as the standard treatment for bilateral severe to profound HL, who does not benefit from conventional Hearing Aid (HA).^2^, ^3^, ^4^ Candidacy for implantation usually reserved for deaf children with better auditory and speech outcome. However, 30%–40% of deaf children have additional disabilities.^5^, ^6^, ^7^ This group of children often has stormy infanthood which lead to delayed referral for HA and CI. Due to the Multiple Disabilities (MD) and hearing impairment; they often have poor speech perception and poor language skills. Therefore, providing amplification for children with MD has been a challenge. The complex process involved in cochlear implantation includes from candidate’s selection, audiological and speech evaluation process, counselling for patients and guardian, the surgical intervention and long-term post-implantation rehabilitation. Another major issue is when the children have inner ear malformation, which traditionally is contraindicated for implantation. However, recent study by Abdullah A et al. (2020) has proven that they showed favourable outcomes comparable to their implanted peers without congenital inner ear malformation.^8^

Children with multiple disabilities and hearing impairment are often overlooked as a potential CI candidate as the outcome varies and spoken communication is often unlikely.^9^ The candidacy for implantation has expanded over the past several years, and an increasing number of deaf children with various disabilities are receiving CI.^5^, ^6^, ^7^ Furthermore, many studies reported positive impacts of CI on children with MD in term of communication, development, and quality of life for not only the children but also the families.^10^, ^11^, ^12^, ^13^, ^14^ Although these population will have lower audiological and speech outcome compared to their peers without additional disability, it is important to keep in mind that they may obtain benefit and improvement in their quality of life with CI.

Child Development Centre (CDC) at our centre used the Diagnostic and Statistical Manual of Mental Disorders (DSM-V) to classify neurodevelopmental disabilities, which includes Global Developmental Delay (GDD) or Intellectual Disability (ID). Global developmental delay is a diagnosis for children less than 5-year-old with at least two modalities from gross/fine motor, speech/language, cognition, social/personal, or activities of daily living. At the same time, ID is for children more than 5-year-old with significant limitations in intellectual functioning and adaptive behaviour. Most GDD progress to ID, and they can represent one population.^15^ Other disabilities include communication disorders, Autism Spectrum Disorder (ASD), Attention-Deficit Hyperactivity Disorder (ADHD), specific learning disorder, motor disorders and other neurodevelopmental disorders such as syndromes.^15^ Developmental delay and cognitive or behavioural disorders are well recognized as poor prognostic factors for language and communication development after implantation. In countries with limited resources, careful selection of CI candidates with the best prognosis post-implantation to achieve language and communication skills are required. This limits the possible improvement in communication and quality of life in children with MD. To date, there is no standardized guidelines to assess CI outcomes in this population. This study aims to determine the benefits of cochlear implantation in children with MD in terms of their audiological outcomes, speech performance, and quality of life. Our hypothesises are, children with MD would have improvement in auditory, speech and language outcomes; and all parents of children with MD would report improvement in child’s quality of life after cochlear implantation.

## Methods

### Design and participants

**Figure 1:**
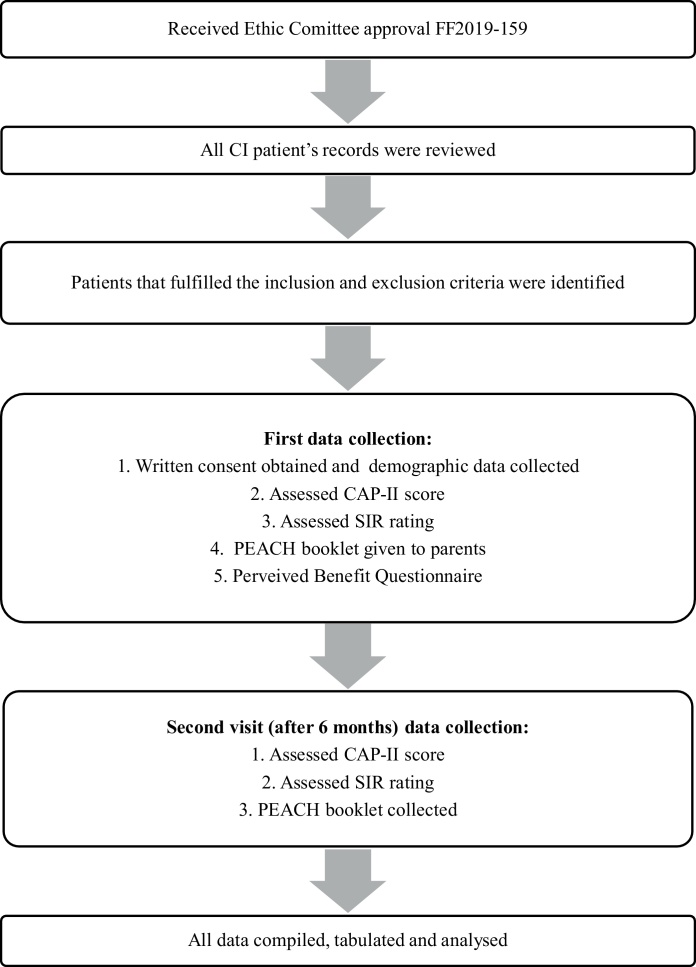
Flowchart of the study.

This is a cross-sectional study conducted from January 2019 till December 2020 and within the duration, 31 families with prelingual hearing impaired children less than 12-year-old were identified from our centre Cochlear Implant Registry. This study received approval from our university Research and Medical Ethic Committee. During the study, authors reviewed the medical chart of the recruited patients then assessment of child’s speech and audiological outcome were done during the first visit of data collection and reassessed 6-months later during second visit as shown in the flowchart (Fig. 1). All children who have used cochlear implant for at least 6-months and were diagnosed with at least one neurodevelopmental disability based on Diagnostic and Statistical Manual of Mental Disorder-V (DSM-V) were recruited.

### Instruments

Categories of Auditory Performance-II (CAP-II) scoring system was used to assess the children’s auditory skills during first data collection and reassess 6-months later at second data collection. Categories of Auditory Performance-II is reproducible, easy to administer, and non-language or race based. It consists of 10 scores which reflects the children’s daily auditory performance, the lowest being score (0) – no awareness to environmental sounds/voice and highest achievable score is (9) – use of telephone with an unknown speaker in an unpredictable context. Parents were also given the Parent’s Evaluation of Aural/Oral Performance of Children (PEACH) diary and were asked to observe their child’s auditory and oral behaviour for a duration of 1-week, then document responses for each item in the booklet with as many examples as possible. Parent’s Evaluation of Aural/Oral Performance of Children diary uses parent’s observation to measure the functional auditory performance of a child and is useful to assess effectiveness of amplification in daily life, independent of child’s age and duration of amplification. During the second visit, the booklet was returned and interview with parents were arranged to review and to clarify any unsure informations. Then, each response to a question was scored on a five-point rating scale ranging from 0 to 4. Never (0): Did not demonstrate any auditory response or no example given; Seldom (1): If auditory response occurred 1%‒25% of the time or 1‒2 examples given; Sometimes (2): If auditory response occurred 26%‒50% of the time or 3‒4 examples given; Often (3): If auditory response occurred 51%‒75% of the time or 5‒6 examples given; Always (4): If auditory response occurred more than 75% of the time or more than 7 examples given. Speech performance was assessed using Speech Intelligibility Rating (SIR) scale. It consists of 5 rating scale from the lowest category or score of 1 when no intelligible speech or recognisable words to the highest score of 5 when connected speech is intelligible to all listeners. The SIR was reassessed twice during the first visit and 6-months later. The Perceived Benefit Questionnaire (PBQ) by S. Wiley et al. (2005) is specifically developed to ascertain quality of life of deaf children with MD post implantation, from the parents’ perspective.^7^ The questionnaire consists of issues observed in daily life of a child, with five types of parents’ response: much improved (5); improved (4); no change (3); worse (2) or much worse (1).

### Statistical analysis

All data was tabulated and analysed using The Statistical Package for the Social Sciences (SPSS) version 26. Since the data were not normally distributed, non-parametric statistical analyses were used.

## Results

### Descriptive data

A total of 31 children and their parents were recruited in this study. Most of the informant were mothers. The children comprised of 17 girls and 14 boys, with mean age of 2.84 years (range 1.25 to 10 years) at time of CI switch on. The mean age was 7.0 years at the first interview (range of 2.2–12.6 years). All 31 children in this study were diagnosed with Global Developmental Delay (GDD) and 10 (32.3%) of them have 2 disabilities while 1 (3.2%) child was diagnosed with 3 disabilities. Table 1 showed the types of disabilities found in this study population.

**Table 1:** Characteristic of the children’s disabilities.

Neurodevelopmental disability	n	(%)
Global Developmental Delay (GDD)/ID	31	100
GDD alone: 20		
GDD with other disability: 11		
Syndromes	6	19.5
Down: 2		
CHARGE: 1		
Phelan-Mcdermid: 1		
Pendred: 1		
VACTERL: 1		
Autism Spectrum Disorder (ASD)	3	9.7
Cerebral Palsy (CP)	1	3.2
Attention Deficit Hyperactivity Disorder (ADHD)	1	3.2

To facilitate the analysis of this study, the children were further divided into 3 groups based on hearing age of 0–2 years (n = 11, 35.5%), 2–5 years (n = 7, 22.6%) and 5–10 years (n = 13, 41.9%). All children had severe to profound or profound hearing loss prior to implantation. All children were compliant in using the CI device with majority (87%) consistently used the device throughout their waking hours.

All children had undergone Magnetic Radiological Imaging (MRI) and Computed Tomography (CT) scans prior to implantation as part of the CI work up. Table 2(a) and (b) showed the anomalies found in these scans, respectively. There were 8 (25.8%) children with multiple radiological anomalies and only 3 (9.7%) of them have normal radiological findings.

**Table 2:** MRI anomalies and CT anomalies.

(a) MRI anomalies		
MRI	n	(%)
Congenital infection:	15	48.4
TORCHES: CMV (8), Rubella (1)		
Unknown: 6		
Other MRI finding:	10	32.3
(Ventriculomegaly, cyst, others)		
No MRI anomalies	6	19.3

(b) CT anomalies		
CT	n	(%)
Cochlear nerve hypoplasia	5	16.1
Incomplete partition of cochlea type 2	3	9.7
Semicircular canal dysplasia	3	9.7
Large vestibular aqueduct	2	6.5
Ossicular anomaly	1	3.2
No CT anomalies	17	54.8

### Auditory performance outcomes

**Figure 2:**
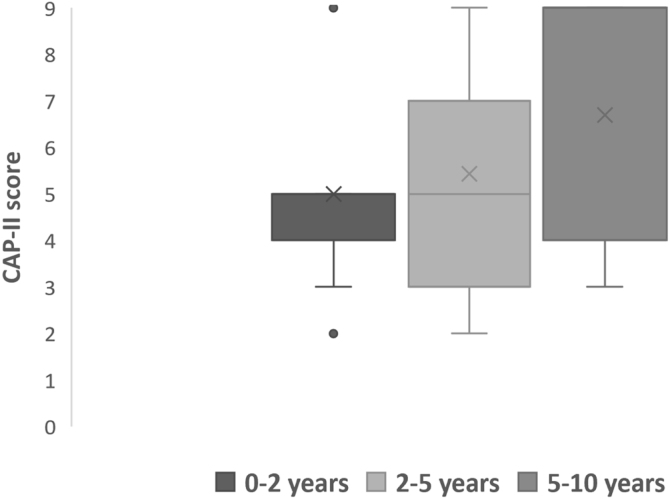
Boxplot of CAP-II score against hearing age.

**Figure 3:**
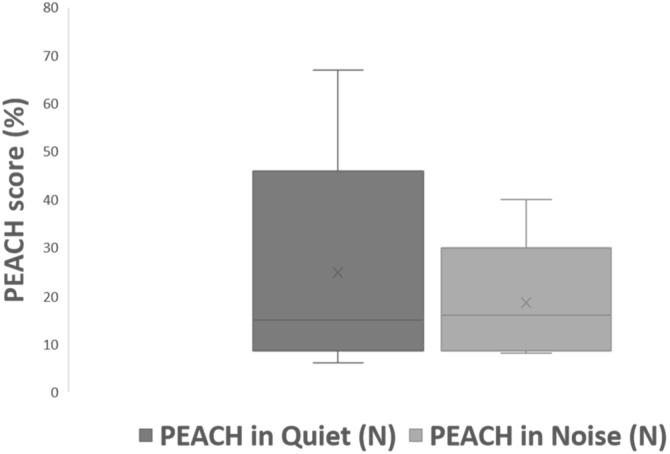
PEACH score (%) in quiet and noisy environment.

**Figure 4:**
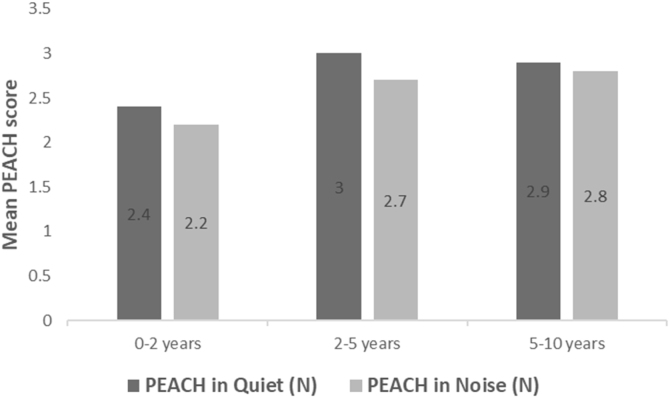
Mean PEACH score against hearing age.

All subjects in this study showed improvement of CAP-II score and then mean value increased with hearing age (Fig. 2). Wilcoxon signed rank test showed significant improvement of CAP-II score from first to second assessment with p-value of 0.001. Fig. 3 showed the mean PEACH score in quiet (24.8%) (IQR = 37.5) is higher than in noise (18.6%) (IQR = 21.5); while Fig. 4 showed mean PEACH score also improved with increased hearing age. For further analysis, we performed Chi-Square test to check relation of GDD alone or GDD with other disability (GDD+), and the ability to achieve CAP-II score of more than 4 (discrimination of speech sound without lip reading). However, there was no significant difference among these two subgroups (*p* = 1.00) (Table 3).

**Table 3:** Chi-Square of GDD alone (GDD) and GDD with other Disability (GDD+) with CAP-II score of more than 4.

	CAP-II		Total	χ^2^	*p*
	<4	≥4			
	n (%)	n (%)	n (%)		
GDD+	4 (36.4)	7 (63.6)	**11** (100)	0.00	1.000
GDD	6 (30)	14 (70)	**20** (100)		

### Speech outcomes

**Figure 5:**
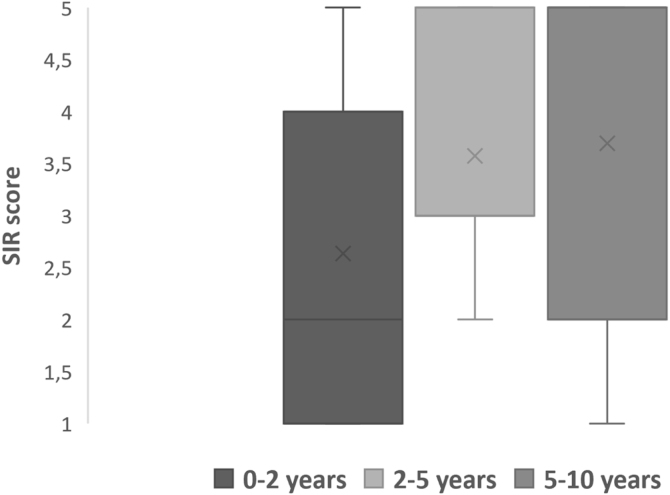
SIR score against hearing age.

Post implantation mode of communications were divided into verbal, non-verbal and total communication. Total communication means incorporation of all means of communication mode such as gestures, fingerspelling, body language, lipreading and speech. After implantation, we observed more than half (64.5%) of the children were able to achieve verbal communication skills (Table 4). Speech Intelligibility Rating (SIR) category scale also improved with increased hearing age (Fig. 5) and Wilcoxon signed rank test showed significant improvement of SIR with *p*-value of 0.002.

**Table 4:** Mode of communication after implantation.

	n	(%)
Verbal:	20	64.55
Oral language: 12 (38.75)		
Oral language + gesture: 8 (25.8)		
Non-verbal:	7	22.55
Sign language: 1 (3.2)		
Gesture: 6 (19.35)		
Total communication	4	12.9

### Quality of life

All parents unanimously agreed that CI was beneficial to their children, no one gave a score of worse from the questionnaire (Table 5). More than 70% of the parents reported improvement in their child’s environmental sound awareness, improved interaction and improved in vocal skill. More than half (67.8%) of the parents reported reduction in family stress level after child’s implantation.

**Table 5:** The perceived benefits Questionnaire as reported by parents.

Perceived benefits	Much improved	Improved	No change	Worse, much worse
	n (%)	n (%)	n (%)	n (%)
1. While my child is wearing the implant, he/she appears more comfortable/relaxed.	13 (42)	7 (54.8)	1 (3.2)	0 (0)
2. My child exhibits less frustration with communication.	13 (42)	15 (48.4)	3 (9.6)	0 (0)
3. My child is more interactive	22 (71)	6 (19.4)	3 (9.6)	0 (0)
4. My child is more vocal	21 (67.8)	7 (22.6)	3 (9.6)	0 (0)
5. My child is more aware of his/her surroundings	24 (77.4)	7 (22.6)	0 (0)	0 (0)
6. My child is accepting of wearing the device.	14 (45.2)	16 (51.6)	1 (3.2)	0 (0)
7. My child’s level of stress in new situations is:	4 (12.9)	15 (48.4)	12 (38.7)	0 (0)
8. My child is able to be soothed by my voice when upset	5 (16.1)	21 (67.8)	5 (16.1)	0 (0)
9. My child is able to alert to warning signals (fire alarm, etc.)	24 (77.4)	7 (22.6)	0 (0)	0 (0)
10. My child is able to responds to auditory commands.	16 (51.7)	12 (38.7)	3 (9.6)	0 (0)
11. The amount of stress felt by our family due to our child’s hearing impairment has:	6 (19.4)	21 (67.8)	4 (12.9)	0 (0)
12. My child is better able to express wants and needs	14 (45.2)	10 (32.2)	7 (22.6)	0 (0)
13. My child’s level of independence has:	9 (29)	18 (58.1)	4 (12.9)	0 (0)
14. My child participate more with our family	13 (42)	14 (45.2)	4 (12.9)	0 (0)
15. My child’s ability to produce speech has:	14 (45.2)	10 (32.2)	7 (22.6)	0 (0)

## Discussion

The presence of additional disabilities in hearing impaired children makes it difficult to predict the audiological and speech outcomes after CI. Nonetheless, all our patients with multiple disabilities showed benefits in terms of audiological outcomes, speech performance and improved in quality of life after cochlear implantation. This study showed GDD is the most common neurodevelopmental disabilities, mainly caused by congenital CMV infection. Similar findings were reported by Edwards et al. (2012) and Tamer et al. (2018).^10^, ^11^, ^12^, ^13^ Children with GDD in this study showed ability to achieve significant CAP-II score more than 4 and there was significant improvement with increased hearing age. This correlates with study by Youm et al. (2012), who reported significant CAP score (mean of 4) in children with ID, 12-months after CI and Birman et al. (2013) that reported 52% of children with developmental delay achieved mean CAP-II score of 5 after 12-months post CI.^16^, ^17^

Our study showed that PEACH score in quiet is better than in noise and there was improvement of mean PEACH score with increasing hearing age. This is similar to findings by Quar et al. (2011) that overall, PEACH score increased as a function to hearing age and Goh et al. (2018), who reported of higher median (87.5%) PEACH score in quiet compared to in noise (85%).^2^, ^18^

Although speech acquisition was deemed unlikely in children with MD, our study showed 64.5% of children with GDD were able to achieve verbal communication and there was significant improvement when hearing age increased. Furthermore, some children with MD have the potential to achieve the maximum score for CAP-II and SIR. Although those with more severe disabilities have little to no speech development, but they developed more audiological awareness which allow them to communicate and interact with caretaker and families.

Wiley et al. (2006) similarly cited in their study that children with MD made communication progress post CI and they have significant SIR score improvement after CI but not sufficient to keep up in term of their age.^7^ Nikolopolous et al. (2008) stated that 70% of children with MD in their study reached connected intelligibility (SIR score 5) after 5-years of CI, but the speech quality differed from child to child.^19^ Besides that, Lachowska et al. (2016) reported that ASD patients do progress from behavioural to verbal communication (vocalization with varies pitch and few short words) after implantation, which is significant for the children and their families.^20^

Overall, parents perceived multiple benefits and gains received by their child after implantation in auditory performance, interaction, communication, quality of life and speech development. Most of children with MD have other therapies and follow up; so the improvement in their hearing and communication allows the child to participate in their treatment and well-being. Wiley et al. (2006) and Berettini et al. (2008) showed similar result of perceived improvement (96%‒100%), speech production 74%.^6^, ^7^ Speaker et al. (2018) showed 84.6% parents reported CI as a positive influence on the child’s quality of life.^21^ Cila et al. (2018) also reported that in general, families are satisfied with CI.^3^ We found that majority children in this study consistently wearing the device and showed progression in the communication skills. The families that participated also very motivated and consistent in their child rehabilitation as they continued their child therapies and rehabilitation despite the recent Coronavirus Disease (COVID-19) pandemic. They outsourced to private or online session and dedicated time at home with the child. These occurs as they experienced the improvement and benefits of CI in their child, and they worked with many professional to attain specific goal or task for the child to achieve.

Authors felt that data from this study is useful as it is from developing country. Since majority of publications are from developed countries, we hope that this outcomes give the perspective of a nation with somewhat fewer resources compared to the developed or first world countries. It is valuable to understand the spectrum of outcomes in different settings. The outcomes were multimodal and add dimension to different aspects of functioning and from various perspectives, including professionals and parents.

## Conclusion

Cochlear implantation had shown benefits in children with multiple disabilities in this study. Outcome measures should not only focus on auditory and speech performances but the improvement in quality of life as reported by parents. Therefore, this group of children should be assessed thoroughly by the CI team and paediatrician before decision for implantation is made with appropriate expectations discussed.

## Funding and conflict of interests

This manuscript has no prior publication and did not receive any financial support from any source. There is no conflict of interest. There is no reproduction of pre-published information/material in this article.

## Conflicts of interest

The authors declare no conflicts of interest.

## References

[1] (2021). World Report on Hearing.

[2] Goh B.S., Fadzilah N., Abdullah A., Othman B.F., Umat C. (2018). Long-term outcomes of Universiti Kebangsaan Malaysia cochlear implant program among paediatric implantees. Intern J Pediatr Otorhinolaryngol.

[3] Umat C., Abdul What N.H., Che Ross S., Goh B.S. (2018). Quality of life of parents and siblings of children with cochlear implants. J Otol.

[4] Archbold S.M., Lutman M.E., Gregory S., O’Neill C., Nikolopoulos T.P. (2002). Parents and their deaf child: their perceptions three years after cochlear implantation. Deafness Educ Int.

[5] Hamzavi J., Baumgartner W.D., Egelierler B., Franz P., Schenk B., Gstoettner W. (2000). Follow up of cochlear implanted handicapped children. Intern J Pediatr Otorhinolaryngol.

[6] Berrettini S., Forli F., Genovese E., Santarelli R., Arslan E., Chilosi A.M. (2008). Cochlear implantation in deaf children with associated disabilities: challenges and outcomes. Intern J Audiol.

[7] Wiley S., Jahnke M., Meinzen-Derr J. (2005). Qualitative benefits of cochlear implants in children with multi-handicaps. Intern J Pediatr Otorhinolaryngol.

[8] Abdullah A., Othman I.A., Goh B.S., Umat C., Tyler R. (2020). Auditory performance in early implanted children with cochleovestibular malformation and cochlear nerve deficiency. J Intern Adv Otol.

[9] McCracken W., Turner O. (2012). Deaf children with complex needs: parental experience of access to cochlear implants and ongoing support. Deaf Educ Int.

[10] Edwards L.C., Frost R., Witham F. (2006). Developmental delay and outcomes in paediatric cochlear implantation: implications for candidacy. Intern J Pediatr Otorhinolaryngol.

[11] Meinzen-Derr J., Wiley S., Grether S., Choo D.I. (2011). Children with cochlear implants and developmental disabilities: a language skills study with developmentally matched hearing peers. Res Dev Disabil.

[12] Wakil N., Fitzpatrick E.M., Olds J., Schramm D., Whittingham J.A. (2014). Long-term outcome after cochlear implantation in children with additional developmental disabilities. Intern J Audiol.

[13] Mesallam T.A., Yousef M., Almasaad A. (2018). Auditory and language skills development after cochlear implantation in children with multiple disabilities. Eur Arch Otorhinolaryngol.

[14] Nasralla H.R., Montefusco A.M., Hoshino A.C., Samuel P.A., Magalhães A.T., Goffi-Gomez M.V. (2018). Benefit of cochlear implantation in children with multiple handicaps: parent’s perspective. Intern Arch Otorhinolaryngol.

[15] Regier D.A., Narrow W.E., Clarke D.E., Kraemer H.C., Kuramoto S.J., Kuhl E.A. (2012). DSM-5 field trials in the United States and Canada, Part II: test-retest reliability of selected categorical diagnoses. Am J Psychiatry.

[16] Youm H.Y., Moon I.J., Kim E.Y., Kim B.Y., Cho Y.S., Chung W.H. (2013). The auditory and speech performance of children with intellectual disability after cochlear implantation. Acta Otolaryngol.

[17] Birman C.S., Elliott E.J., Gibson W.P.R. (2012). Pediatric cochlear implants. Otol Neurotol.

[18] Quar T.K., Ching T.Y.C., Mukari S.Z.M.S. (2012). Newall P Parents’ evaluation of aural/oral performance of children (PEACH) scale in the Malay language: data for normal-hearing children. Intern J Audiol.

[19] Nikolopoulos T.P., Archbold S.M., Wever C.C., Lloyd H. (2008). Speech production in deaf implanted children with additional disabilities, and comparison with age-equivalent implanted children without such disorders. Intern J Pediatr Otorhinolaryngol.

[20] Lachowska M., Pastuszka A., Łukaszewicz-Moszyńska Z., Mikołajewska L., Niemczyk K. (2016). Cochlear implantation in autistic children with profound sensorineural hearing loss. Braz J Otorhinolaryngol.

[21] Speaker R.B., Roberston J., Simoes-Franklin C., Glynn F., Walshe P., Viani L. (2018). Quality of life outcomes in cochlear implantation of children with profound and multiple learning disability. Cochlear Implants Intern.

